# Isolated intestinal type angioedema due to ACE‐inhibitor therapy

**DOI:** 10.1002/ccr3.925

**Published:** 2017-03-31

**Authors:** Stephen Palmquist, Benji Mathews

**Affiliations:** ^1^ University of Minnesota Medical School‐Duluth CampusDuluthMN; ^2^ Health Partners & University of Minnesota Medical SchoolSt. PaulMN

**Keywords:** Accurate and timely diagnosis, ACE‐inhibitor side effects, intestinal angioedema, lisinopril‐induced small bowel edema

## Abstract

A 42‐year‐old woman presents with abdominal pain after taking her first dose of lisinopril. Visceral angioedema was diagnosed based on clinical suspicion and abdominal computed tomography (CT). Awareness of this rare side effect of a common medication is key to avoid delays in diagnosis and unnecessary procedures.

## Introduction

Angioedema is a rare, but well‐known side effect of ACE inhibitors causing facial, tongue, and lip swelling in 0.1–1.0% of patients 1. Intestinal type angioedema is a less reported form of angioedema, causing swelling of the small bowel with associated nausea, vomiting, and/or diarrhea. This report is on a 42‐year‐old African American woman who developed isolated intestinal type angioedema with nausea and vomiting beginning 12 h after starting lisinopril.

## Case Presentation

A 42‐year‐old African American woman with a history of hypertension, morbid obesity, and obstructive sleep apnea presented to the emergency department with a 12‐hour history of left‐sided abdominal pain, nausea, and vomiting. At presentation, her medications included hydrochlorothiazide, acetaminophen, ibuprofen, and lisinopril. The patient is married, has three children, and has never smoked, consumed alcohol, or used illicit drugs.

The patient had travelled to Ghana for 5 days, 1 month prior to presentation. She denies any symptoms during or since her trip until her current symptoms started. She was prescribed lisinopril for hypertension, and her first dose of lisinopril 5 mg was 12 h before the onset of her symptoms. She had never taken an ACE inhibitor (ACEi) or angiotensin II receptor antagonist (ARB) before.

On presentation, she had crampy abdominal pain that radiated to the left side of her abdomen. Her pain was constant and increasing but did wax and wane in severity. She also had persistent nausea with one bout of emesis. She had one nonbloody bowel movement of normal caliber within 24 h of presenting and denied any recent diarrhea, constipation, or recent sick contacts.

Vitals at presentation included a blood pressure of 187/122, pulse of 81, temperature of 36.7°C, respirations of 20, and an O_2_ saturation of 100% on room air. Physical examination showed a patient that appeared uncomfortable and at times sitting up and writhing in bed with her eyes closed. Her abdomen was tender to palpation in the left epigastric area and left mid‐abdomen, with mild left suprapubic tenderness. She also had hyperactive bowel sounds but no rebound tenderness or significant guarding.

**Figure 1:**
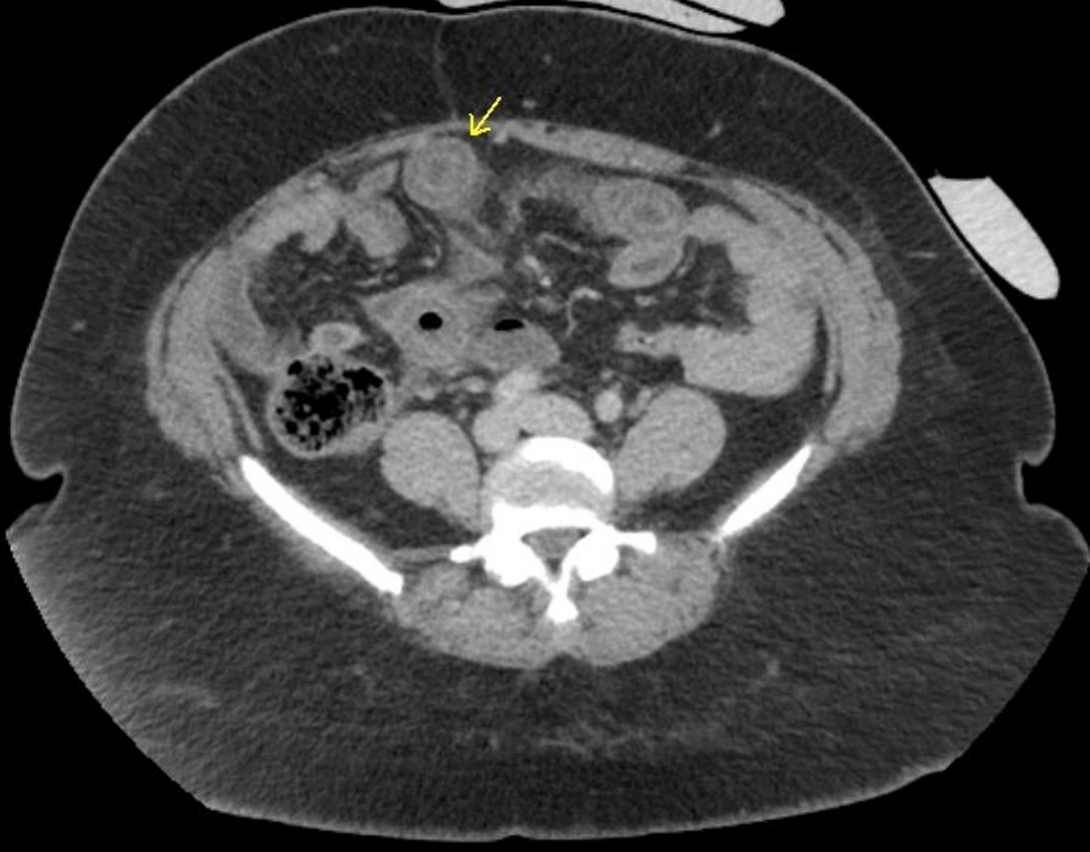
Axial contrast‐enhanced CT images demonstrated thickening of the bowel wall with submucosal edema and all layers of the bowel delineated. (Yellow arrows).

**Figure 2:**
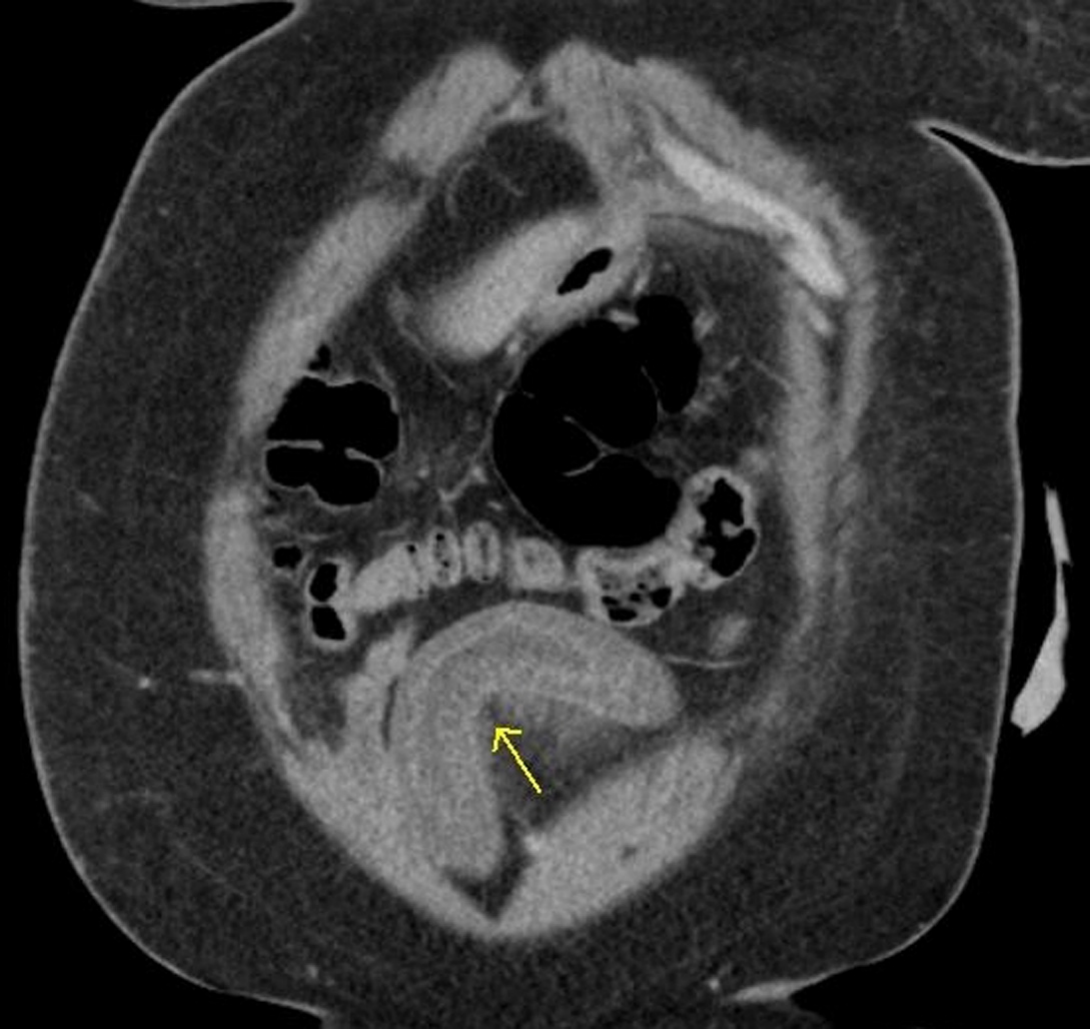
Coronal contrast‐enhanced CT image demonstrated thickening of the bowel wall with submucosal edema and all layers of the bowel delineated. (Yellow arrows).

In the emergency department, the patient received intravenous fluids, as well as pain and nausea medications. The laboratories showed an unremarkable CBC. Her C1 esterase inhibitor functional assay was within normal limits along with a normal C4 concentration making type I and type II hereditary angioedema unlikely. Stool studies were negative for infectious diseases. CT of the abdomen showed segmented target‐like enhancement of a dilated loop of ileum with relatively high attenuation ascites (Figs 1 and 2). The initial differential included inflammatory bowel disease, infectious enteritis, vasculitis intramural hemorrhage, and lymphoma.

Given her admission clinical and radiographic findings, drug‐induced visceral angioedema due to ACEi was suspected. Lisinopril was discontinued and the patient's abdominal symptoms resolved completely in 48 h. The patient was discharged to home on 5 mg of amlodipine for blood pressure control and instructed to follow‐up with her primary care provider. On follow‐up 2 and 8 weeks later, her symptoms had not returned.

## Discussion

Angioedema occurs in 0.1–0.77% of patients taking ACE inhibitors 2. It usually manifests as swelling of the face, tongue, and lips, and in rare cases, the gastrointestinal wall. Angioedema of the face and visceral regions occurs within the first week of taking an ACE inhibitor in 59–60% of cases, so the timing of onset is similar regardless of body area involved 3.

Since angioedema is less obvious when involving the intestine, it presents a diagnostic challenge. A MEDLINE search was carried out to elucidate key characteristics. Using search terms “intestinal angioedema,” “visceral angioedema,” “ACE‐inhibitor side effects,” and the names of ACE inhibitors such as lisinopril, captopril, enalapril, benazepril, a list of articles were compiled from 1980 to 1 August 2016. Thirty‐four individual reports of ACE inhibitor induced visceral angioedema fit the criteria in question 4, 5, 6, 7, 8, 9, 10, 11, 12, 13, 14, 15, 16, 17, 18, 19, 20, 21, 22, 23, 24, 25, 26, 27, 28, 29, 30, 31, 32. Signature features to assist in diagnosing ACEi‐induced intestinal type angioedema are denoted in Table 1 and are discussed below.

**Table 1:** Characteristics of ACEI‐induced isolated angioedema

*n *=* *34	*n* (%)
Gender	
Male	5 (14.7)
Female	29 (85.3)
Ethnicity	
White	5 (14.7)
African American	9 (26.5)
Asian	1 (2.9)
Unreported	19 (55.9)
Age	
Average age (Years)	49.5, SD = 15.3
Presenting symptoms	
Abdominal pain	34 (100)
Emesis	26 (76.5)
Diarrhea	16 (47.1)
ACEI patient was taking	
Lisinopril	19 (55.9)
Enalapril	5 (14.7)
Captopril	3 (8.8)
Benazepril	2 (5.9)
Ramipril	2 (5.9)
Fosinopril	2 (5.9)
Temocapril	1 (2.9)

From the information gathered, there are several trends throughout the case reports. There is a disproportionate number of women 4, 5, 6, 8, 9, 10, 11, 12, 13, 14, 15, 16, 17, 19, 20, 21, 22, 23, 24, 27, 28, 29, 30, 32 affected (85%) compared to men 7, 18, 25, 26, 31. The age of patients ranged from 26 to 92 years, with the average age being 49.5 years (SD = 15.3) 4, 5, 6, 7, 8, 9, 10, 11, 12, 13, 14, 15, 16, 17, 18, 20, 21, 22, 23, 24, 25, 26, 27, 28, 29, 30, 31, 32 Ethnicity was only reported in 15 cases, nine of which reported the patient being African American 4, 6, 8, 10, 22, 25.

The particular ACE inhibitor implicated in the case reports was wide ranging and included lisinopril (56%) 4, 5, 6, 8, 10, 12, 15, 16, 18, 19, 21, 24, 28, 29, 31, 32, enalapril (15%) 9, 13, 14, 27 captopril (9%) 7, 25, 30, benazepril (6%) 11, 22, ramipril (6%) 17, 20, fosinopril (6%) 16, 23, and temocapril (3%) 26. The cases also reported reactions at both high and low doses of ACE inhibitor.

Presenting symptoms tended to be nonspecific with all patients having abdominal pain 4, 5, 6, 7, 8, 9, 10, 11, 12, 13, 14, 15, 16, 17, 18, 19, 20, 21, 22, 23, 24, 25, 26, 27, 28, 29, 30, 31, 32, 76% having emesis 4, 6, 7, 8, 10, 11, 12, 13, 14, 15, 16, 19, 21, 22, 23, 24, 25, 26, 27, 29, 30, 31, 32 and 47% having diarrhea 4, 6, 7, 8, 9, 12, 15, 19, 23, 24, 25, 26, 27, 29, 31, 32. Another finding was that laboratory results, including CBC, were overwhelmingly normal. Sixteen cases reported running a C1 esterase inhibitor functional assay, and all sixteen cases reported as this laboratory was within normal limits 4, 7, 10, 11, 13, 14, 15, 16, 18, 20, 23, 24, 25, 28, 29. After identification, ACEi‐induced visceral angioedema patients had rapid recovery times. The average time to resolution of symptoms was 1.9 days (SD = 1.5) as reported by 16 of the case reports 5, 7, 8, 9, 10, 11, 12, 13, 14, 15, 19, 21, 22, 23, 24.

There were 10 reported colonoscopies and/or esophagogastroduodenoscopies as part of the workup 4, 11, 12, 14, 16, 19, 23, 27, 32. Another 12 abdominal surgeries were reported as well 7, 10, 16, 21, 26, 29, 30. Consistent through the cases was the value of an abdominal CT scan in making the diagnosis. Cases in which an abdominal CT was obtained, the scans showed small bowel edema 4, 5, 6, 7, 8, 9, 10, 11, 12, 13, 14, 15, 16, 18, 19, 20, 21, 22, 23, 24, 26, 28, 32.

In this case, the patient had many signature findings including a middle‐aged woman taking an ACEi, African American ethnicity, abdominal pain without infectious findings, CT findings of bowel‐wall thickening, normal C1‐esterase inhibitor level and resolution of symptoms after cessation of medication. All of these findings in our patient made us confident in our diagnosis of isolated intestinal type angioedema.

## Conclusion

ACE‐inhibitor‐induced visceral angioedema should be considered in the differential diagnosis of abdominal pain. Isolated intestinal angioedema secondary to ACE inhibitors is a rare side effect of ACEi causing severe abdominal pain. Awareness is important for early suspicion and will help in avoiding delays in diagnosis, unnecessary testing, and considerable morbidity. Visceral angioedema should receive the same level of attention by providers as the classic facial angioedema. Signature findings include a middle‐aged female patient on an ACE inhibitor presenting with abdominal pain without infectious etiologies and an abdominal CT scan showing small bowel edema. Cessation of all ACE inhibitors and symptomatic treatment leads to resolution of symptoms.

## Authorship

SP: contributed to the article by co‐writing the article and conducting the literature review with data analysis. BM: contributed to the article by co‐writing and editing the article.

## Conflict of Interest

None declared.
